# Time Course of Symptoms in Normal-Pressure Hydrocephalus: A Systematic Review

**DOI:** 10.3390/diagnostics15141778

**Published:** 2025-07-14

**Authors:** Bekir Rovčanin, Ibrahim Omerhodžić, Adem Nuhović, Emir Begagić, Nevena Mahmutbegović, Hakija Bečulić, Haso Sefo, Enra Mehmedika-Suljić, Almir Džurlić, Mirza Pojskić

**Affiliations:** 1 Department of Neurosurgery, Clinical Center University of Sarajevo, Bolnička 25, 71000 Sarajevo, Bosnia and Herzegovina; bekir_rovcanin@hotmail.com (B.R.); ibrahimomerhodzic74@gmail.com (I.O.); haso_sefo@hotmail.com (H.S.); almir_dz86@hotmail.com (A.D.); 2 Faculty of Medicine, University of Sarajevo, Čekaluša 90, 71000 Sarajevo, Bosnia and Herzegovina; enra.suljic@gmail.com; 3 Department of Infectious Disease, Clinical Center University of Sarajevo, Bolnička 25, 71000 Sarajevo, Bosnia and Herzegovina; ademnuhovic@gmail.com; 4 Department of Neurosurgery, Cantonal Hospital Zenica, Crkvice 67, 72000 Zenica, Bosnia and Herzegovina; begagicem@gmail.com (E.B.); dr_beculichakija@hotmail.com (H.B.); 5 Department of Neurology, University Clinical Center Sarajevo, Bolnička 25, 71000 Sarajevo, Bosnia and Herzegovina; nevenaradulovic@hotmail.com; 6 Department of Anatomy, School of Medicine, University of Zenica, Travnička 1, 72000 Zenica, Bosnia and Herzegovina; 7 Sarajevo Medical School, University Sarajevo School of Science and Technology, Hrasnička cesta 3a, 71000 Sarajevo, Bosnia and Herzegovina; 8 Department of Neurosurgery, University Hospital Marburg, 35033 Marburg, Germany

**Keywords:** idiopathic normal-pressure hydrocephalus, ventriculoperitoneal shunt, symptom duration, postoperative outcomes, prognostic factors

## Abstract

**Background and Objectives:** Idiopathic normal-pressure hydrocephalus (NPH) is a treatable, but diagnostically challenging condition in the elderly marked by gait disturbance, cognitive decline, and urinary incontinence. Ventriculoperitoneal (VP) shunting is effective, but the prognostic significance of symptom duration before surgery remains unclear. This systematic review evaluates symptom duration in NPH patients with postoperative outcomes. **Methods:** A systematic search of PubMed, Scopus, and Embase was conducted per PRISMA guidelines. Studies were included if they assessed clinical or radiological outcomes of VP shunting in adult NPH patients, reported symptom duration, and had a follow-up of at least one month. Clinical outcomes (MMSE, TUG, NPH score) were qualitatively analyzed due to study heterogeneity. **Results:** Twenty-four studies comprising 1169 patients were included (mean age: 72.45 years; mean symptom duration: 33.04 months). Most studies reported clinical improvement after VP shunting. However, few directly evaluated the effect of symptom duration, yielding inconsistent findings: some suggested better outcomes with shorter symptom duration, while others found no clear correlation. Larger studies often lacked conclusive data, and no randomized controlled trials were identified. **Conclusions:** VP shunting remains an effective intervention for NPH; however, evidence supporting the predictive value of preoperative symptom length is inconclusive. This review highlights the need for standardized diagnostic protocols and larger prospective studies to clarify this association and optimize surgical timing.

## 1. Introduction

Normal-pressure hydrocephalus (NPH) is a clinical syndrome prevalent in up to 2.9% of the elderly population characterized by gait disturbances, cognitive decline, and urinary dysfunction [1,2]. Its diagnosis poses challenges due to overlapping clinical features with other neurological conditions common in elderly individuals, such as Parkinson’s disease and Alzheimer’s disease [3,4]. NPH can arise secondary to various pathological processes, including inflammation, traumatic brain injury, or meningitis; however, in approximately 50% of cases, it is considered idiopathic with no discernible underlying cause [1].

NPH should be considered in the differential diagnosis when adults present with unexplained, gradual-onset gait disturbance, urinary incontinence, and cognitive impairment. Typically, gait disturbance is the earliest and most prominent symptom, often accompanied by milder cognitive deficits that may improve with treatment. Unlike Alzheimer’s disease, which generally involves more severe, irreversible memory loss without early gait abnormalities, NPH’s gait issues are prominent early on [3,4].

In contrast to Parkinson’s disease, NPH usually does not cause tremors or muscle rigidity; however, some patients may exhibit movement disorders such as akinesia, tremulousness, hypertonia, or hyperkinesia. While both NPH and Parkinson’s can cause gait disturbances, Parkinson’s patients often show rigidity, slowed movements (bradykinesia), and a narrow-based gait, whereas NPH typically presents with a broad-based gait. Additional signs of NPH may include reduced facial expressiveness, difficulty swallowing (dysphagia), excessive salivation, and orthostatic hypotension [1,2,3,4].

Neuroimaging techniques like MRI or CT scans combined with cerebrospinal fluid (CSF) drainage tests are valuable tools in evaluating these symptoms and establishing the treatment strategy.

Despite its distinct neuroradiological presentation, there remains a lack of universally accepted diagnostic criteria. The Japanese Society of Normal Pressure Hydrocephalus published guidelines in 2021 to aid management, yet diagnosing NPH remains clinically intricate [5]. Epidemiological studies have employed various scales to assess symptom severity, yet definitive thresholds distinguishing normal from impaired function remain undefined in current guidelines.

Accurate diagnosis is crucial, given that 70–80% of NPH patients may benefit from surgical intervention; however, the criteria for selecting candidates for shunt surgery remain ambiguous. Earlier studies suggested that delaying CSF shunting after symptom onset decreased the likelihood of improvement post-surgery [1]. Conversely, recent viewpoints suggest that delayed response to treatment, rather than symptom duration, may account for unsuccessful outcomes following surgery [6]. The efficacy of shunting in NPH patients has been questioned due to the absence of robust randomized controlled trials (RCTs) in this domain. The recent literature suggests that the duration of clinical symptoms may serve as a prognostic indicator of surgical outcomes [7,8,9,10,11]. We conducted a systematic review of the literature to elucidate these issues and assess the predictive value of preoperative symptom duration in ventriculoperitoneal (VP) shunting for NPH.

## 2. Materials and Methods

### 2.1. Study Design and Registration

The methodology of this systematic review adhered to the Preferred Reporting Items for Systematic Reviews and Meta-Analyses (PRISMA) guidelines and PRISMA checklist, provided in Appendix A.

The study was registered on the Open Science Framework (OSF) (identifier OSF-REGISTRATIONS-8GD9K-V1).

### 2.2. Search Strategy

The search strategy, based on the PICOS framework, focused on adults diagnosed with NPH as the population (P) and VP shunt surgery as the intervention (I). No specific comparison group (C) or outcomes (O) were defined, allowing for a broad inclusion of studies. The search was restricted to original research studies (S) to ensure the inclusion of primary data on the effectiveness and outcomes of VP shunt surgery in NPH patients (Table 1). A systematic search of the electronic databases PubMed, Scopus, and Embase was conducted on 31 October 2023. Detailed search syntax is provided in Appendix B.

### 2.3. Inclusion and Exclusion Criteria

The inclusion criteria for the study were: (1) studies evaluating clinical or radiological outcomes following shunt surgery; (2) adult patients diagnosed with NPH; (3) documentation of pre- and post-shunt test scores; (4) insertion of a VP shunt; (5) reporting of symptom duration prior to VP shunt insertion; and (6) a follow-up period exceeding one month.

The exclusion criteria were: (1) non-English publications; (2) review articles; (3) congress proceedings; (4) books and book chapters; and (5) studies lacking data of interest, including (a) studies where outcomes could not be tracked both pre- and post-surgery, (b) studies reporting modified NPH tests, (c) studies utilizing NPH tests that were not among the specified outcomes, (d) reports that did not specify the shunt type or where the shunt was not a VP shunt, (e) lack of access to the full text, and (f) studies on secondary VP shunt procedures.

### 2.4. Study Selection

Titles and abstracts of identified articles were independently screened by three reviewers (N.M., B.R., A.N.) to determine eligibility for full-text review. In cases of disagreement, senior reviewers (I.O. and H.B.) were consulted to resolve inclusion decisions. Given the limited number of studies with large populations, efforts were made to include as many relevant studies as possible. For studies that were potentially eligible, but lacked complete data, corresponding authors were contacted via email to request additional information. Additionally, reference lists of included studies were reviewed to identify further relevant articles.

**Figure 1:**
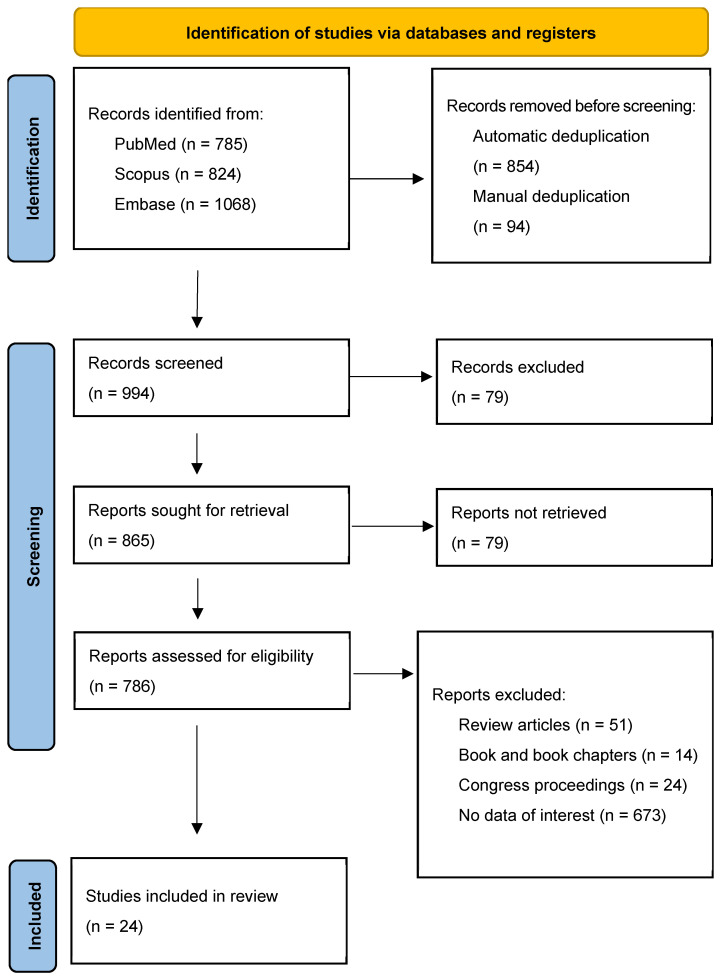
PRISMA flow diagram.

A total of 2677 records were identified across PubMed (*n* = 785), Scopus (*n* = 824), and Embase (*n* = 1068). After automatic deduplication using Endnote (*n* = 854) and manual deduplication (*n* = 94), 944 records remained for screening. Of these, 79 reports could not be retrieved, leaving 865 reports for eligibility assessment. Following the exclusion of review articles (*n* = 51), book chapters (*n* = 14), congress proceedings (*n* = 24), and studies lacking data of interest (*n* = 752), 24 studies were included in the final review (Figure 1).

### 2.5. Data Extraction, Quality Assessment, and Statistical Analysis

The general characteristics extracted from the studies included the country of origin, year of publication, study design, duration of symptoms, and sample demographics (age, sex, follow-up period). Primary outcome measures assessed pre- and postoperatively were the Mini-Mental State Examination (MMSE), timed up and go (TUG) test measured in steps and seconds, the 10 m walk test measured in steps and seconds, NPH grading scale and score, and the Evans index. Inclusion criteria for studies required the presence of at least one of these outcome measures.

Extracted data encompassed primary outcomes, publication year, number of patients, percentage of male participants, duration of symptoms, follow-up period, ethnicity of the study population, and study design. While some studies did not explicitly investigate the timing of VP surgery, individual patient data reported in these studies warranted their inclusion due to the scarcity of larger studies.

To evaluate the methodological rigor and risk of bias in the studies included in this systematic review, we employed the Newcastle–Ottawa Scale (NOS), a widely accepted tool for appraising the quality of non-randomized studies. The NOS assesses three key domains: selection of study groups (maximum 4 stars), comparability of cohorts (maximum 2 stars), and outcome assessment (maximum 3 stars), yielding a total score out of 9. Each of the 24 studies included in our review was independently evaluated using the NOS for studies. Where study information was ambiguous or incomplete, ratings were conservatively assigned to avoid overestimation of methodological quality.

A qualitative synthesis was performed, but due to the heterogeneity of the studies, quantitative synthesis was not feasible.

## 3. Results

### 3.1. Sample Characteristics

The study included a total of 1169 patients, of whom 637 (57%) were male (Table 2). Across 24 studies included, the overall weighted mean age was 72.45 years and the weighted mean duration of symptoms was 33.04 months. The mean follow-up period after shunt surgery was 7.05 months, ranging from 12 to 54 months. Most of the studies included in the analysis were prospectively designed. No randomized controlled trials were identified.

### 3.2. Effects of Symptom Duration

Only four studies provided conclusions regarding the correlation between symptom duration and clinical outcomes. Foss T. et al. found no significant difference in symptom duration between patients who responded to ventriculoperitoneal shunting (VPS) and those who did not [28]. Poca MA et al. similarly concluded that there was no statistically significant association between symptom duration and outcomes. In contrast, George R. et al. reported that patients with symptom durations of two years or less experienced greater improvement in dementia, with the Mini-Mental State Examination (MMSE) scores increasing from a mean of 23.5 before VPS to 28 after three months [31]. Elisabeth S. et al. also found that shorter symptom durations were associated with improved MMSE scores, with a mean increase from 24 before VPS to 25 after six months. However, all these studies had small samples, except for the study by Solana E. et al., which included more than 180 participants [25].

Nineteen studies did not explicitly report the effects of symptom duration on clinical outcomes. Among these studies, most reported improvements in clinical outcomes following VPS, with only two outcomes across two studies showing no improvement or worsening. The largest study included in the review, conducted by Simon Agerskov G. et al., involved 429 participants, but did not report any conclusions regarding the impact of symptom duration on outcomes [13].

### 3.3. Follow-Up and Clinical Outcome Data

Clinical outcomes were extensively documented, with 17 studies using the MMSE as a basic assessment tool for patients with idiopathic normal-pressure hydrocephalus. Fifteen of these studies reported MMSE scores during the follow-up period, with all but one study showing an increase in MMSE scores after VPS. In studies with a three-month follow-up, the range of MMSE improvement was between 0 and 4.5 points. One study with a one-month follow-up reported an improvement of 0.5 points. Studies with a six-month follow-up showed an improvement range of 1 to 2.59 points, while those with a twelve-month follow-up reported improvements ranging from 0.3 to 2 points (Table 3).

Foss T. et al. reported that shunt non-responders were more often female and younger, with no difference in symptom duration compared to responders [28]. Similarly, Poca MA et al. found no significant association between symptom duration and other outcomes [31]. In contrast, Razay G. et al. observed that shorter symptom duration was linked to greater improvement in dementia functioning (CIBIC-plus ratings of 2.0 vs. 3.0, *p* = 0.03), while Elisabeth S. et al. found that a shorter illness duration independently predicted better MMSE and Purdue Pegboard scores, with women exhibiting more improvement in verbal memory [12,25]. Additionally, those patients with four traditionally poor prognostic markers (idiopathic form, cortical atrophy, disease evolution > 12 months, and dementia) were identified as having less favorable outcomes [34]. Table 4 and Table 5 present follow-up measures of 10 m/s and steps) and Evans index and NPH scores regarding symptom duration across studies.

The outcome domain varied across studies depending on follow-up duration and the use of validated clinical endpoints (e.g., MMSE, TUG, NPH score). Although many studies demonstrated pre- and post-shunt comparisons, few included blinded or independent outcome assessment. Additionally, short follow-up periods in several studies limited their ability to capture long-term effects or late complications of VP shunting.

### 3.4. Quality Assessment of Included Studies

Based on the NOS in Table 6, risk of bias was rated as moderate in 23 out of 24 included studies (95.8%), while only a single study met criteria for low risk of bias. The overall moderate risk profile across the dataset is largely attributable to uniformly low scores in the comparability domain. Specifically, all 24 studies scored only 1 out of a possible 2 points, indicating that no study adequately adjusted for more than one key confounding variable—such as age, sex, vascular risk factors, or baseline functional status—which are essential for controlling bias in observational research. In the selection domain, most studies (*n* = 19) scored 3 out of 4 points, reflecting generally acceptable cohort selection and NPH diagnosis criteria. Only one study [13] achieved the maximum of 4 points, while four studies [11,18,30,32,36,37] scored only 2 points, indicating potential weaknesses in patient selection or diagnostic ascertainment. In the outcome domain, 21 studies scored 2 out of 3 points, while only two studies [30,32] scored 1 point, suggesting either inadequate follow-up duration or insufficiently described outcome assessment. Overall, 23 of 24 studies (95.8%) were classified as having a moderate risk of bias, and none achieved full scores across all NOS domains. These findings indicate considerable methodological limitations, particularly regarding confounder control and study comparability.

## 4. Discussion

In this systematic review, we did not find a randomized clinical trial with normal-pressure hydrocephalus patients investigating the duration of symptoms and its impact on postoperative clinical outcomes. Regarding the duration of NPH symptoms before ventriculoperitoneal shunt surgery and its role in clinical outcomes after surgery, we were unable to find a clear conclusion.

We found that the weighted mean duration of NPH symptoms in our patients before undergoing VPS was 33.04 months. Our results show that a ventriculoperitoneal shunt is effective in patients with NPH.

Traditionally, symptom duration has been considered a poor factor for predicting unfavorable surgery outcomes in NPH patients [31]. Meier and Miethke found that the duration of symptoms before shunt surgery is shorter than 12 months, which results in better outcomes [34]. However, some studies do not support this conclusion. In one study, compared with patients shunted early, delayed surgical intervention in the long term did showed no difference in clinical outcomes [8]. Also, studies conducted from 2016 to 2019 did not find the effect of symptom duration on clinical outcomes in NPH patients [38].

One recent study has shown that delayed treatment of 6 to 24 months of NPH can result in a higher mortality rate. But this study had major methodological limitations in terms of study design and difference in age between two groups, which can affect results [39]. Also, a recent study published in 2020 reported a statistically significant cutoff of 9.5 months of duration of symptoms for the best clinical effect and improved knowledge about the treatment of NPH [40]. In our review, we also reached similar contrary conclusions. Elisabeth S et al. conducted a study on 185 patients with mean symptom duration of 24 months and observed that shorter illness duration was independently associated with an improvement in some clinical outcomes, like MMSE scores. Poca MA et al. did not observe any statistically significant association between symptom duration and any of the other outcome measures [25,31].

Foss T. et al. reported that shunt non-responders were more often female and younger than shunt responders, but the duration of symptoms was not different between the groups [28]. Also, other authors like Andrén and Yamada did not observe any association between the duration of clinical symptoms and postoperative clinical outcome; however, Yamada pointed out that patients diagnosed with NPH should undergo shunt surgery before symptoms become severe and negatively influence quality of life [41,42].

We considered undertaking a statistical meta-analysis of collected study data, but that was not possible due to study heterogeneity and reported data without unique methodology and study design.

In this study, we found that a ventriculoperitoneal shunt is an effective treatment for NPH without regard to preoperative symptom duration. In terms of clinical outcomes of the MMSE, TUG, 10 m, NPH score, and Evans score, it seems that there is an improvement after VPS, but there is just a short follow-up. Later clinical worsening and deterioration after initial good improvement were reported in many studies. Takeuchi et al. reported that the MMSE, TUG test, and mRS started to deteriorate at a mean of 3 years postoperatively and urinary symptoms 2 years postoperatively. However, in a 4-year follow-up, the mean scores of these tests were still better than preoperative scores [43].

Later deterioration in a subset of patients with initially good treatment effects has been described in many studies with repeated long-term follow-up. On the timed-up-and-go test, MMSE, and mRS, deterioration in mean scores started at 3 years and in urinary symptoms after 2 years. The tendency of decline was more pronounced in the older group. Noteworthily, in all parameters, despite this deterioration, the mean scores at 4 years were still better than the preoperative results [43].

In a recent study, it was found that long-term outcomes in patients with probable NPH treated with VPS were not significantly influenced by age, comorbidities, or responses to CSF sampling. This indicates that these traditional clinical factors may have limited value in predicting postoperative prognosis. Notably, the authors emphasized the importance of preoperative CSF dynamics, particularly aqueductal flow acceleration observed on dynamic brain MRI, which showed a significant correlation with neurological improvement at 12 and 18 months after surgery. These findings underscore the role of radiological assessment as a prognostic factor in patients considered suitable candidates for VPS surgery, while subtraction tests warrant further investigation. Despite the extended follow-up period, statistical significance was only observed up to 18 months post-surgery, highlighting the difficulty in identifying reliable long-term prognostic factors in patients with NPH [44]. One study assessed 30 patients at baseline and 2 and 15 days after 24 h extended lumbar drainage, measuring motor and cognitive performance through tests such as the TUG, MMSE, MoCA, and FAB. The authors emphasized the importance of conducting multiple assessments following CSF drainage in patients with suspected NPH, as the response to treatment may become more apparent over time. They highlighted the need for a delayed response assessment, especially for cognition. These findings highlight that delayed evaluations can provide more reliable insights into a patient’s potential benefit from shunt surgery, underscoring the importance of timing in preoperative assessment protocols [45].

In a review of patients treated between 1999 and 2013 following VPS surgery with adjustable gravitational valves for idiopathic NPH, about 20% of patients experienced secondary clinical deterioration approximately 2.7 years after initial improvement. The review’s authors emphasized the importance of long-term follow-up after VPS. Key risk factors identified included older age at shunting, the development of neurodegenerative diseases, and episodes of overdrainage requiring higher-pressure adjustments. These findings underscore the importance of long-term follow-up and aggressive shunt management to maximize sustained clinical benefit, as roughly one-quarter of patients with secondary decline can regain stability through pressure adjustments or shunt revision, pointing to the need for vigilant monitoring to optimize long-term outcomes in NPH patients [14].

Delayed surgical intervention in NPH patients was associated with significant clinical decline. In patients waiting for surgery, the total NPH score fell from a median of 45 to 37, with marked worsening in mRS, MMSE, and nearly all NPH subdomains (continence decline was only at trend level). Consequently, the postoperative benefit in delayed cases did not surpass the preoperative status at diagnosis. In a registry of 3.007 NPH patients (SHQR, 2004–2019), waiting times were grouped as ≤3 months, 3.1–5.9 months, and ≥6 months. At three months post-surgery, 57% of those with ≤3-month delays achieved a significant (≥5 point) improvement on the modified NPH scale versus 52% and 46% in the longer-delay groups (*p* = 0.0115). Similar trends were observed at 12 months (61%, 52%, and 51%; *p* = 0.0536). Improvements in MMSE and TUG were consistent across groups, highlighting that shunt surgery within three months of decision-making is critical for optimal outcomes [15]. Benveniste and Sur observed that delayed symptom progression may be more common in older age; however, 80% of the patients had objective clinical improvement after shunting [16].

### Study Limitations

The reliance on retrospective studies in this systematic review introduces inherent biases, including selection bias and recall bias, due to potential issues with data collection that may be incomplete or inconsistent. Many of the studies had small samples, limiting their findings’ generalizability, as these cohorts may not adequately represent the broader population with NPH and may inflate the risk of random error. Additionally, the absence of randomized controlled trials (RCTs) constrains the ability to draw causal inferences, as the lack of randomization allows confounding factors to influence outcomes, complicating the establishment of clear relationships between interventions and results. Variability in diagnostic criteria for NPH across studies may lead to heterogeneous patient populations, further complicating outcome comparisons and affecting the validity of the synthesized results. Furthermore, selective reporting of outcomes in some studies may bias conclusions regarding treatment efficacy and safety. Follow-up periods also varied significantly among studies, potentially resulting in inconsistent outcomes, as short durations may fail to capture long-term effects or complications associated with NPH. The diversity of treatment protocols employed across studies raises concerns about the consistency of interventions, while variations in demographic and clinical characteristics of the patients examined may adversely affect the generalizability of the findings to the wider NPH population. One of the key limitations of this study is the presence of substantial qualitative heterogeneity among the included studies. In particular, the lack of control or comparator groups in most studies combined with inconsistent reporting of outcome measures prevented the possibility of conducting a meta-analysis. Given these limitations, it is strongly recommended that future studies on NPH adopt standardized reporting practices and include appropriate control groups to enable more robust quantitative synthesis.

## 5. Conclusions

In conclusion, while our findings affirm that VPS remains a treatment modality for NPH, they simultaneously underscore the necessity of additional research to better understand how symptom duration affects surgical outcomes. A unified approach in study design, including larger prospective trials and standardized diagnostic criteria, is essential for elucidating the complexities concerning NPH treatment. In light of our findings, we strongly advocate for timely intervention following diagnosis to optimize patient outcomes and quality of life in this challenging clinical landscape.

## Figures and Tables

**Table 1:** PICOS search strategy.

Acronym	Search Strategy
P (*population or problem*)	Adults diagnosed with idiopathic normal-pressure hydrocephalus (NPH)
I (*intervention*)	Ventriculoperitoneal (VP) shunt surgery
C (*comparison*)	None
O (*outcome*)	None
S (*study design*)	Original research studies

**Table 2:** Baseline characteristics of included studies.

Study	Year	*n*	Male (*n*)	Mean Age (Years)	Follow-Up Intervals (Months)	Country	Type of Study	Duration of Symptoms (Months)
Razay G. et al. [12]	2019	62	39	77.6	3, 6, 12, >12 months	Australia	Retrospective	24
Agerskov G. et al. [13]	2018	429	266	71	3 and 6	Sweden	Retrospective	44
Wetzel C. et al. [14]	2018	32	21	71.2	3 and 6	Germany	Prospective	22.9
Tullberg M. et al. [15]	2008	18	11	68	3	Sweden	Prospective	40
Yang L. et al. [16]	2017	27	17	71.1	3 and 6	China	Prospective	12
Yang L. et al. [16]	2017	31	20	71.4	3 and 6	China	Prospective	12
Mendes GAS et al. [17]	2017	29	18	73.9	12 and more	Spain	Prospective	19.9
Mendes GAS et al. [17]	2017	26	15	71.5	12	Brazil	Prospective	27.33
Ziegelitz D. et al. [18]	2015	5	4	69	3	Sweden	Prospective	22
Ringstad G. et al. [19]	2015	17	9	74	12	Norway	Prospective	24
Ziegelitz D. et al. [20]	2015	15	8	71	3	Sweden	Prospective	36
Jurcoane A. et al. [21]	2014	12	9	74	24	Germany	Prospective	25
Lundin F. et al. [22]	2013	20		73	3	Sweden	Prospective	36
Oliveira MF et al. [23]	2012	24	13	77.1	12	Brazil	Prospective	27.3
Lundin F. et al. [24]	2012	14	8	74	3	Sweden	Prospective	37
Solana E. et al. [25]	2012	185	111	73.96	6	Spain	Prospective	24
Katzen H. et al. [26]	2011	12	4	74.92	6	USA	Prospective	26.96
Tisell M. et al. [11]	2011	7		75	3 and 6	Sweden	Prospective	36
Razay G. et al. [12]	2008	18	9	76.4	3 to 4	Australia	Prospective	55
Nakayama T. et al. [27]	2007	8	5	74.9	1	Japan	Prospective	27.6
Foss T. et al. [28]	2006	27	8	72	6 and 9	Norway	Prospective	30
Eide PK et Brean A. [29]	2006	40	21	75	12	Norway	Prospective	24
Eide PK [30]	2005	39	14	71	12	Norway	Retrospective	27.6
Poca MA et al. [31]	2005	12	8	74.08	6	Spain	Prospective	40
Eide PK et Sorteberg [32]	2005	19	8	68	12	Norway	Prospective	30
Poca MA et al. [31]	2004	43	30	71.1	6	Spain	Prospective	35.6

**Table 3:** Values of Mini-Mental Scale Examination and TUG (s) and (steps) index in follow-up.

**Study**	***n***	**Duration of Symptoms (Months)**	**MMSE (0 m, 3 m, 6 m, 12 m)**	**TUG (s) (0 m, 3 m, 6 m, 12 m)**	**TUGs (Steps) (0 m, 3 m, 6 m, 12 m)**
Agerskov G. et al. [13]	429	44	0 m: 25; 3 m: 27.2	0 m: 17; 3 m: 12	N/A
Wetzel C. et al. [14]	32	22.9	0 m: 24.5; 3 m: 26.1; 6 m: 26.8	N/A	N/A
Oliveira MF et al. [27]	24	27.3	0 m: 19	0 m: 21; 3 m: 41	0 m: 35.77
I.Illán-Gala I. et al. [33]	29	19.9	0 m: 25	3 m: 25.3	N/A
Lundin F. et al. [29]	20	36	0 m: 27; 3 m: 27	0 m: 20; 3 m: 13.5	6 m: 26.5; 12 m: 19
Lundin F. et al. [29]	14	37	0 m: 28	0 m: 19.5; 3 m: 12	6 m: 29.5; 12 m: 17
Solana E. et al. [25]	185	24	0 m: 24; 6 m: 25	N/A	N/A
Katzen H. et al. [30]	12	26.96	0 m: 24.5; 6 m: 25.58	N/A	N/A
Razay G. et al. [12]	18	54	0 m: 23.5; 6 m: 28	0 m: 17; 3 m: 13	N/A
Tisell M. et al. [11]	18	40	0 m: 23.3; 6 m: 25.9	N/A	N/A
Foss T. et al. [28]	27	30	0 m: 25; 6 m: 26+	N/A	N/A
Poca MA et al. [25]	43	35.6	0 m: 21.33; 6 m: 22.56	N/A	N/A
Jurcoane A. et al. [24]	12	25	0 m: 26.3; 6 m: 27.6	N/A	N/A
Tisell M. et al. [11]	7	36	0 m: 22.5	0 m: 33.6; 3 m: 18; 6 m: 22	N/A
Poca MA et al. [31]	12	40	0 m: 19.83; 6 m: 22.42	N/A	N/A
Mendes GAS et al. [21]	26	27.33	0 m: 20	0 m: 22; 3 m: 38.7	6 m: 27.6
Nakayama T. et al. [32]	8	27.6	0 m: 21.3; 1 m: 21.8	N/A	N/A

N/A—not available.

**Table 4:** Values of 10 m (s) and (steps) and Evans index in follow-up.

**Study**	***n***	**Duration of Symptoms** **(Months)**	**10 m (s)** **(0 m, 3 m, 6 m, 12 m)**	**10 m (Steps)** **(0 m, 3 m, 6 m, 12 m)**	**EVANS Index** **(0 m, 3 m, 6 m, 12 m)**
Lundin F. et al. [26]	20	36	0 m: 16.5	0 m: 11.5	-
Lundin F. et al. [29]	14	37	0 m: 15	0 m: 9	0 m: 0.38; 3 m: 0.37
Ringstad G. et al. [23]	18	54	0 m: 17.4	0 m: 14.2	N/A
Tullberg M. et al. [20]	18	40	0 m: 23.4	0 m: 12.6	N/A
Jurcoane A. et al. [24]	12	25	0 m: 9.4	6 m: 8.4	N/A
Tisell M. et al. [11]	7	36	0 m: 25.3; 3 m: 33	N/A	N/A
Oliveira MF et al. [27]	24	27.3	N/A	N/A	0 m: 0.37; 12 m: 0.33
Ziegelitz D. et al. [22]	15	36	N/A	N/A	0 m: 0.4; 3 m: 0.37
Ziegelitz D. et al. [35]	5	22	N/A	N/A	0 m: 0.42; 3 m: 0.41
Poca MA et al. [31]	12	40	N/A	N/A	0 m: 0.38; 12 m: 0.33
Mendes GAS et al. [21]	26	27.33	N/A	N/A	0 m: 0.38; 12 m: 0.34

**Table 5:** NPH scores in included studies.

**Study**	***n***	**Duration of Symptoms (Months)**	**NPH Score** **(0 m, 3 m, 6 m, 12 m)**
Yang L. et al. [19]	27	27	0 m: 10.15; 3 m: 11.56; 6 m: 11.93
Yang L. et al. [19]	31	12	0 m: 10.39; 3 m: 11.84; 6 m: 12.58
Ringstad G. et al. [23]	17	24	0 m: 10; 12 m: 12
Ziegelitz D. et al. [22]	15	36	0 m: 48; 3 m: 68
Ziegelitz D. et al. [35]	5	22	0 m: 65; 3 m: 62
Eide PK et Brean A. [36]	40	24	0 m: 10; 12 m: 12
Eide PK [18]	39	27.6	0 m: 9; 12 m: 12
Poca MA et al. [31]	43	35.6	0 m: 9.21; 6 m: 12.42
Poca MA et al. [31]	12	40	0 m: 8.75; 6 m: 12.67
Eide PK et Sorteberg [37]	19	30	0 m: 9; 12 m: 11

**Table 6:** Newcastle–Ottawa Scale (NOS) assessment of methodological quality.

**Study (Author, Year, Reference)**	**Selection**	**Comparability**	**Outcome**	**Total**	**Risk of Bias**
Razay G. et al. [12]	3	1	2	6	Moderate risk
Agerskov G. et al. [13]	4	2	2	8	Low risk
Wetzel C. et al. [17]	3	1	2	6	Moderate risk
Tullberg M. et al. [20]	3	1	2	6	Moderate risk
Yang L. et al. [19]	3	1	2	6	Moderate risk
Mendes GAS et al. [21]	3	1	2	6	Moderate risk
Ziegelitz D. et al. [21]	3	1	2	6	Moderate risk
Ringstad G. et al. [23]	3	1	2	6	Moderate risk
Jurcoane A.et al. [24]	3	1	2	6	Moderate risk
Lundin F. et al. [26]	3	1	2	6	Moderate risk
Oliveira MF et al. [27]	3	1	2	6	Moderate risk
Solana E. et al. [25]	3	1	2	6	Moderate risk
Katzen H. et al. [30]	2	1	1	4	Moderate risk
Tisell M. et al. [11]	2	1	2	5	Moderate risk
Nakayama T. et al. [32]	2	1	1	4	Moderate risk
Foss T. et al. [28]	2	1	2	5	Moderate risk
Eide PK et Brean A. [36]	3	1	2	6	Moderate risk
Eide PK [18]	2	1	2	5	Moderate risk
Poca MA et al. [31]	3	1	2	6	Moderate risk
Eide PK et Sorteberg [37]	2	1	2	5	Moderate risk
Illán-Gala I. et al. [33]	3	1	2	6	Moderate risk
Ziegelitz D. et al. [22]	3	1	2	6	Moderate risk

## Data Availability

Data are contained within the article.

## Appendix A

**Section/Topic**	**#**	**Checklist Item**	**Reported on Page #**
TITLE			
Title	1	Identify the report as a systematic review, meta-analysis, or both.	1
ABSTRACT			
Structured summary	2	Provide a structured summary including, as applicable: background; objectives; data sources; study eligibility criteria, participants, and interventions; study appraisal and synthesis methods; results; limitations; conclusions and implications of key findings; systematic review registration number.	1
**Introduction**			
Rationale	3	Describe the rationale for the review in the context of what is already known.	2
Objectives	4	Provide an explicit statement of questions being addressed with reference to participants, interventions, comparisons, outcomes, and study design (PICOS).	2
**Methods**			
Protocol and registration	5	Indicate if a review protocol exists, if and where it can be accessed (e.g., web address), and, if available, provide registration information including registration number.	2
Eligibility criteria	6	Specify study characteristics (e.g., PICOS, length of follow-up) and report characteristics (e.g., years considered, language, publication status) used as criteria for eligibility, giving rationale.	2
Information sources	7	Describe all information sources (e.g., databases with dates of coverage, contact with study authors to identify additional studies) in the search and date last searched.	3
Search	8	Present full electronic search strategy for at least one database, including any limits used, such that it could be repeated.	Appendix B
Study selection	9	State the process for selecting studies (i.e., screening, eligibility, included in systematic review, and, if applicable, included in the meta-analysis).	3
Data collection process	10	Describe method of data extraction from reports (e.g., piloted forms, independently, in duplicate) and any processes for obtaining and confirming data from investigators.	3
Data items	11	List and define all variables for which data were sought (e.g., PICOS, funding sources) and any assumptions and simplifications made.	3
Risk of bias in individual studies	12	Describe methods used for assessing risk of bias of individual studies (including specification of whether this was done at the study or outcome level), and how this information is to be used in any data synthesis.	N/A
Summary measures	13	State the principal summary measures (e.g., risk ratio, difference in means).	N/A
Synthesis of results	14	Describe the methods of handling data and combining results of studies, if done, including measures of consistency (e.g., I^2^) for each meta-analysis.	N/A
Risk of bias across studies	15	Specify any assessment of risk of bias that may affect the cumulative evidence (e.g., publication bias, selective reporting within studies).	Table 6
Additional analyses	16	Describe methods of additional analyses (e.g., sensitivity or subgroup analyses, meta-regression), if done, indicating which were pre-specified.	N/A
**Results**			
Study selection	17	Give numbers of studies screened, assessed for eligibility, and included in the review, with reasons for exclusions at each stage, ideally with a flow diagram.	Figure 1
Study characteristics	18	For each study, present characteristics for which data were extracted (e.g., study size, PICOS, follow-up period) and provide the citations.	3
Risk of bias within studies	19	Present data on risk of bias of each study and, if available, any outcome level assessment (see item 12).	N/A
Results of individual studies	20	For all outcomes considered (benefits or harms), present, for each study: (a) simple summary data for each intervention group (b) effect estimates and confidence intervals, ideally with a forest plot.	Table 1, Table 2 and Table 3
Synthesis of results	21	Present results of each meta-analysis done, including confidence intervals and measures of consistency.	N/A
Risk of bias across studies	22	Present results of any assessment of risk of bias across studies (see Item 15).	N/A
Additional analysis	23	Give results of additional analyses, if done (e.g., sensitivity or subgroup analyses, meta-regression [see Item 16]).	N/A
**Discussion**			
Summary of evidence	24	Summarize the main findings including the strength of evidence for each main outcome; consider their relevance to key groups (e.g., healthcare providers, users, and policymakers).	4–8
Limitations	25	Discuss limitations at study and outcome level (e.g., risk of bias), and at review level (e.g., incomplete retrieval of identified research, reporting bias).	10
Conclusions	26	Provide a general interpretation of the results in the context of other evidence, and implications for future research.	11
**Funding**			
Funding	27	Describe sources of funding for the systematic review and other support (e.g., supply of data); role of funders for the systematic review.	11

## Appendix B

**Database**	**Medline (PubMed)**
Filter	None
Search details	(((((((((normal) AND pressure) AND hydrocephalus)) AND (((((hydrocephalus, normal pressure[MeSH Terms]) OR normal pressure hydrocephalus[MeSH Terms]) OR normal pressure hydrocephalus) OR mph normal pressure hydrocephalus[MeSH Terms]) OR mph))) AND (((((shunting) OR ventriculoperitoneal shunt) OR shunt, ventriculoperitoneal[MeSH Terms]) OR ventriculoperitoneal shunt[MeSH Terms]) OR ventriculoperitoneal))) AND (((adult[MeSH Terms]) OR aged[MeSH Terms]) OR adult)) AND English[lang].” Only studies published in the English language were selected.
**Database**	**Scopus**
Filter	None
Search details	(TITLE-ABS-KEY(normal AND pressure AND hydrocephalus) AND (TITLE-ABS-KEY(normal pressure hydrocephalus) OR TITLE-ABS-KEY(mph normal pressure hydrocephalus)) AND (TITLE-ABS-KEY(shunting) OR TITLE-ABS-KEY(ventriculoperitoneal shunt) OR TITLE-ABS-KEY(ventriculoperitoneal)) AND (TITLE-ABS-KEY(adult) OR TITLE-ABS-KEY(aged)) AND (LIMIT-TO(LANGUAGE, “English”)))
**Database**	**Embase**
Filter	None
Search details	(normal AND pressure AND hydrocephalus) AND (“normal pressure hydrocephalus”/exp OR “mph normal pressure hydrocephalus”) AND (“shunting”/exp OR “ventriculoperitoneal shunt”/exp OR “ventriculoperitoneal”) AND (“adult”/exp OR “aged”/exp) AND [english]/lim

## References

[1] McGirt M.J., Woodworth G., Coon A.L., Thomas G., Williams M.A., Rigamonti D. (2005). Diagnosis, treatment, and analysis of long-term outcomes in idiopathic normal-pressure hydrocephalus. Neurosurgery.

[2] Martín-Láez R., Caballero-Arzapalo H., López-Menéndez L.Á., Arango-Lasprilla J.C., Vázquez-Barquero A. (2015). Epidemiology of Idiopathic Normal Pressure Hydrocephalus: A Systematic Review of the Literature. World Neurosurg..

[3] Relkin N., Marmarou A., Klinge P., Bergsneider M., Black P.M. (2005). Diagnosing idiopathic normal-pressure hydrocephalus. Neurosurgery.

[4] Williams M.A., Malm J. (2016). Diagnosis and Treatment of Idiopathic Normal Pressure Hydrocephalus. Contin. Minneap. Minn..

[5] Nakajima M., Yamada S., Miyajima M., Ishii K., Kuriyama N., Kazui H., Kanemoto H., Suehiro T., Yoshiyama K., Kameda M. (2021). Guidelines for Management of Idiopathic Normal Pressure Hydrocephalus (Third Edition): Endorsed by the Japanese Society of Normal Pressure Hydrocephalus. Neurol. Med. Chir..

[6] Kazui H., Miyajima M., Mori E., Ishikawa M. (2015). SINPHONI-2 Investigators. Lumboperitoneal shunt surgery for idiopathic normal pressure hydrocephalus (SINPHONI-2): An open-label randomised trial. Lancet Neurol..

[7] Andrén K., Wikkelsø C., Tisell M., Hellström P. (2014). Natural course of idiopathic normal pressure hydrocephalus. J. Neurol. Neurosurg. Psychiatry.

[8] Czosnyka Z., Owler B., Keong N., Santarius T., Baledent O., Pickard J.D., Czosnyka M. (2011). Impact of duration of symptoms on CSF dynamics in idiopathic normal pressure hydrocephalus. Acta Neurol. Scand..

[9] Vakili S., Moran D., Hung A., Elder B.D., Jeon L., Fialho H., Sankey E.W., Jusué-Torres I., Goodwin C.R., Lu J. (2016). Timing of surgical treatment for idiopathic normal pressure hydrocephalus: Association between treatment delay and reduced short-term benefit. Neurosurg. Focus.

[10] Klinge P., Hellström P., Tans J., Wikkelsø C. (2012). European iNPH Multicentre Study Group. One-year outcome in the European multicentre study on iNPH. Acta Neurol. Scand..

[11] Tisell M., Tullberg M., Hellström P., Edsbagge M., Högfeldt M., Wikkelsö C. (2011). Shunt surgery in patients with hydrocephalus and white matter changes. J. Neurosurg..

[12] Razay G., Vreugdenhil A., Liddell J. (2009). A prospective study of ventriculo-peritoneal shunting for idiopathic normal pressure hydrocephalus. J. Clin. Neurosci. Off. J. Neurosurg. Soc. Australas..

[13] Agerskov S., Hellström P., Andrén K., Kollén L., Wikkelsö C., Tullberg M. (2018). The phenotype of idiopathic normal pressure hydrocephalus-a single center study of 429 patients. J. Neurol. Sci..

[14] Gutowski P., Rot S., Fritsch M., Meier U., Gölz L., Lemcke J. (2020). Secondary deterioration in patients with normal pressure hydrocephalus after ventriculoperitoneal shunt placement: A proposed algorithm of treatment. Fluids Barriers CNS.

[15] Chidiac C., Sundström N., Tullberg M., Arvidsson L., Olivecrona M. (2022). Waiting time for surgery influences the outcome in idiopathic normal pressure hydrocephalus—A population-based study. Acta Neurochir..

[16] Benveniste R.J., Sur S. (2018). Delayed symptom progression after ventriculoperitoneal shunt placement for normal pressure hydrocephalus. J. Neurol. Sci..

[17] Wetzel C., Goertz L., Schulte A.P., Goldbrunner R., Krischek B. (2018). Minimizing overdrainage with flow-regulated valves—Initial results of a prospective study on idiopathic normal pressure hydrocephalus. Clin. Neurol. Neurosurg..

[18] Eide P.K. (2006). Intracranial pressure parameters in idiopathic normal pressure hydrocephalus patients treated with ventriculo-peritoneal shunts. Acta Neurochir..

[19] Yang L., Wang X., Li Y., Chen L., Yan Z., She L., Dong J. (2017). The Clinical Effect of Postoperative Hyperbaric Oxygen Therapy on Idiopathic Normal Pressure Hydrocephalus: A Retrospective and Comparative Analysis of 61 Patients with Ventriculoperitoneal Shunt. World Neurosurg..

[20] Tullberg M., Blennow K., Månsson J.E., Fredman P., Tisell M., Wikkelsö C. (2008). Cerebrospinal fluid markers before and after shunting in patients with secondary and idiopathic normal pressure hydrocephalus. Cerebrospinal Fluid Res..

[21] Mendes G.A.S., de Oliveira M.F., Pinto F.C.G. (2017). The Timed Up and Go Test as a Diagnostic Criterion in Normal Pressure Hydrocephalus. World Neurosurg..

[22] Ziegelitz D., Arvidsson J., Hellström P., Tullberg M., Wikkelsø C., Starck G. (2016). Pre-and postoperative cerebral blood flow changes in patients with idiopathic normal pressure hydrocephalus measured by computed tomography (CT)-perfusion. J. Cereb. Blood Flow Metab. Off. J. Int. Soc. Cereb. Blood Flow Metab..

[23] Ringstad G., Emblem K.E., Eide P.K. (2016). Phase-contrast magnetic resonance imaging reveals net retrograde aqueductal flow in idiopathic normal pressure hydrocephalus. J. Neurosurg..

[24] Jurcoane A., Keil F., Szelenyi A., Pfeilschifter W., Singer O.C., Hattingen E. (2014). Directional diffusion of corticospinal tract supports therapy decisions in idiopathic normal-pressure hydrocephalus. Neuroradiology.

[25] Solana E., Sahuquillo J., Junqué C., Quintana M., Poca M.A. (2012). Cognitive disturbances and neuropsychological changes after surgical treatment in a cohort of 185 patients with idiopathic normal pressure hydrocephalus. Arch. Clin. Neuropsychol. Off. J. Natl. Acad. Neuropsychol..

[26] Lundin F., Ledin T., Wikkelsø C., Leijon G. (2013). Postural function in idiopathic normal pressure hydrocephalus before and after shunt surgery: A controlled study using computerized dynamic posturography (EquiTest). Clin. Neurol. Neurosurg..

[27] Oliveira MFde Saad F., Reis R.C., Rotta J.M., Pinto F.C.G. (2013). Programmable valve represents an efficient and safe tool in the treatment of idiopathic normal-pressure hydrocephalus patients. Arq. Neuropsiquiatr..

[28] Foss T., Eide P.K., Finset A. (2007). Intracranial pressure parameters in idiopathic normal pressure hydrocephalus patients with or without improvement of cognitive function after shunt treatment. Dement. Geriatr. Cogn. Disord..

[29] Lundin F., Tisell A., Leijon G., Leinhard O.D., Davidsson L., Grönqvist A., Wikkelsø C., Lundberg P. (2013). Preoperative and postoperative 1H-MR spectroscopy changes in frontal deep white matter and the thalamus in idiopathic normal pressure hydrocephalus. J. Neurol. Neurosurg. Psychiatry.

[30] Katzen H., Ravdin L.D., Assuras S., Heros R., Kaplitt M., Schwartz T.H., Fink M., Levin B.E., Relkin N.R. (2011). Postshunt cognitive and functional improvement in idiopathic normal pressure hydrocephalus. Neurosurgery.

[31] Poca M.A., Mataró M., Del Mar Matarín M., Arikan F., Junqué C., Sahuquillo J. (2004). Is the placement of shunts in patients with idiopathic normal-pressure hydrocephalus worth the risk? Results of a study based on continuous monitoring of intracranial pressure. J. Neurosurg..

[32] Nakayama T., Ouchi Y., Yoshikawa E., Sugihara G., Torizuka T., Tanaka K. (2007). Striatal D2 receptor availability after shunting in idiopathic normal pressure hydrocephalus. J. Nucl. Med. Off. Publ. Soc. Nucl. Med..

[33] Illán-Gala I., Pérez-Lucas J., Martín-Montes A., Máñez-Miró J., Arpa J., Ruiz-Ares G. (2017). Evolución a largo plazo de la hidrocefalia crónica del adulto idiopática tratada con válvula de derivación ventrículo-peritoneal. Neurología.

[34] Meier U., Miethke C. (2003). Predictors of outcome in patients with normal-pressure hydrocephalus. J. Clin. Neurosci. Off. J. Neurosurg. Soc. Australas..

[35] Ziegelitz D., Arvidsson J., Hellström P., Tullberg M., Wikkelsø C., Starck G. (2015). In Patients with Idiopathic Normal Pressure Hydrocephalus Postoperative Cerebral Perfusion Changes Measured by Dynamic Susceptibility Contrast Magnetic Resonance Imaging Correlate with Clinical Improvement. J. Comput. Assist. Tomogr..

[36] Eide P.K., Brean A. (2006). Intracranial pulse pressure amplitude levels determined during preoperative assessment of subjects with possible idiopathic normal pressure hydrocephalus. Acta Neurochir..

[37] Eide P.K., Sorteberg W. (2010). Diagnostic intracranial pressure monitoring and surgical management in idiopathic normal pressure hydrocephalus: A 6-year review of 214 patients. Neurosurgery.

[38] Thavarajasingam S.G., El-Khatib M., Rea M., Russo S., Lemcke J., Al-Nusair L., Vajkoczy P. (2021). Clinical predictors of shunt response in the diagnosis and treatment of idiopathic normal pressure hydrocephalus: A systematic review and meta-analysis. Acta Neurochir..

[39] Andrén K., Wikkelsø C., Hellström P., Tullberg M., Jaraj D. (2021). Early shunt surgery improves survival in idiopathic normal pressure hydrocephalus. Eur. J. Neurol..

[40] Krahulik D., Vaverka M., Hrabalek L., Hampl M., Halaj M., Jablonsky J., Langova K. (2020). Ventriculoperitoneal shunt in treating of idiopathic normal pressure hydrocephalus-single-center study. Acta Neurochir..

[41] Andrén K., Wikkelsø C., Laurell K., Kollén L., Hellström P., Tullberg M. (2024). Symptoms and signs did not predict outcome after surgery: A prospective study of 143 patients with idiopathic normal pressure hydrocephalus. J. Neurol..

[42] Yamada S., Kimura T., Jingami N., Masamichi A., Hirai O., Tokuda T., Miyajima M., Kazui H., Mori E., Ishikawa M. (2016). Disability risk or unimproved symptoms following shunt surgery in patients with idiopathic normal-pressure hydrocephalus: Post hoc analysis of SINPHONI-2. J. Neurosurg..

[43] Takeuchi T., Yajima K. (2019). Long-term 4 Years Follow-up Study of 482 Patients Who Underwent Shunting for Idiopathic Normal Pressure Hydrocephalus-Course of Symptoms and Shunt Efficacy Rates Compared by Age Group. Neurol. Med. Chir..

[44] Bianconi A., Colonna S., Minardi M., Di Perna G., Ceroni L., Nico E., Garbossa D., Borgarello S., Cofano F. (2024). Prognostic Factors in Idiopathic Normal Pressure Hydrocephalus Patients After Ventriculo-Peritoneal Shunt: Results from a Single-Institution Observational Cohort Study with Long Term Follow-Up. World Neurosurg..

[45] Caneva S., Hamedani M., Pesaresi A., Mori L., Marzi A., Pellegrino L., Merciadri P., Bianconi A., Zona G., Pardini M. (2025). Beyond early motor response: Longitudinal cognitive and gait assessments after extended lumbar drainage in normal pressure hydrocephalus. Eur. J. Neurol..

